# LM-CycleGAN: Improving Underwater Image Quality Through Learned Perceptual Image Patch Similarity and Multi-Scale Adaptive Fusion Attention

**DOI:** 10.3390/s24237425

**Published:** 2024-11-21

**Authors:** Jiangyan Wu, Guanghui Zhang, Yugang Fan

**Affiliations:** 1 Faculty of Information Engineering and Automation, Kunming University of Science and Technology, Kunming 650500, China; 2 Yunnan Key Laboratory of Intelligent Control and Application, Kunming University of Science and Technology, Kunming 650500, China

**Keywords:** underwater image enhancement, cycle-consistent generative adversarial networks, multi-scale adaptive fusion attention, learned perceptual image patch similarity

## Abstract

The underwater imaging process is often hindered by high noise levels, blurring, and color distortion due to light scattering, absorption, and suspended particles in the water. To address the challenges of image enhancement in complex underwater environments, this paper proposes an underwater image color correction and detail enhancement model based on an improved Cycle-consistent Generative Adversarial Network (CycleGAN), named LPIPS-MAFA CycleGAN (LM-CycleGAN). The model integrates a Multi-scale Adaptive Fusion Attention (MAFA) mechanism into the generator architecture to enhance its ability to perceive image details. At the same time, the Learned Perceptual Image Patch Similarity (LPIPS) is introduced into the loss function to make the training process more focused on the structural information of the image. Experiments conducted on the public datasets UIEB and EUVP demonstrate that LM-CycleGAN achieves significant improvements in Structural Similarity Index (SSIM), Peak Signal-to-Noise Ratio (PSNR), Average Gradient (AG), Underwater Color Image Quality Evaluation (UCIQE), and Underwater Image Quality Measure (UIQM). Moreover, the model excels in color correction and fidelity, successfully avoiding issues such as red checkerboard artifacts and blurred edge details commonly observed in reconstructed images generated by traditional CycleGAN approaches.

## 1. Introduction

Underwater imaging plays a crucial role across various fields, including marine ecology research, underwater archaeology, underwater engineering, and marine resource exploration [1,2,3,4]. However, underwater images often degrade during acquisition and transmission due to multiple factors [5]. For instance, the optical properties of water and suspended particles can cause light scattering and absorption, reducing contrast and color accuracy in underwater images. Additionally, unstable conditions such as water currents and waves can affect the stability of image acquisition, further complicating underwater imaging. Therefore, Underwater Image Enhancement (UIE) is particularly important.

In recent years, traditional and deep learning-based methods have made significant advancements in the field of UIE. However, these methods still exhibit certain limitations when faced with complex underwater environments. On the one hand, physical model-based UIE methods may struggle to obtain sufficient prior information in challenging environments, leading to issues such as color distortion or overcompensation. On the other hand, non-physical model-based methods often rely on fixed parameter settings or heuristic rules, which tend to demonstrate poor generalization capabilities. Although deep learning-based methods have partially addressed these issues, they either fall short in precise feature extraction, suffer from inadequate generalization performance, or typically require a substantial amount of labeled data for training. Therefore, the development of a UIE method that possesses strong generalization capabilities, high robustness, and efficient real-time performance is particularly critical. To this end, this paper proposes a novel model, LM-CycleGAN, with strong adaptability and superior enhancement. Specifically, a Multi-scale Adaptive Fusion Attention (MAFA) mechanism is designed and integrated into the generator, enhancing the generator’s ability to perceive detailed image features. Additionally, the Learned Perceptual Image Patch Similarity (LPIPS) [6] is incorporated into the loss function, effectively introducing deep structural information about the image into the model’s training process. In summary, the main contributions of this study can be outlined as follows:

A Multi-scale Adaptive Fusion Attention Mechanism was designed, enabling multi-scale adaptive fusion across different heads in multi-head attention, significantly enhancing the generator’s capability to extract detailed image features.

The computation of LPIPS was optimized and integrated into the loss function of CycleGAN, improving the model’s ability to assess the similarity between two images and enhancing the quality of image reconstruction results.

The proposed LM-CycleGAN model demonstrated superior performance on the UIEB [7], EUVP [8], and RUIE [9] datasets, validating its effectiveness in the field of UIE.

## 2. Related Work

Depending on the realization principle, underwater image enhancement methods can be mainly classified into two main categories: traditional methods and deep learning-based methods. Among them, the traditional methods are further categorized into physical model-based methods and non-physical model-based methods.

### 2.1. Physical Model-Based Methods

Physical model-based methods construct models by considering image degradation factors in specific environments, thereby reversing or compensating for degradation effects and improving image quality [10,11,12]. For instance, Liu et al. [11] developed a method for nighttime foggy images using a nonlinear and variational Retinex model to estimate illumination and reflectance, effectively removing haze and improving image quality. He et al. [12] introduced the Dark Channel Prior (DCP) algorithm, which clears haze by analyzing dark channel information, enhancing image clarity. Inspired by the successful application of the DCP in removing the “fog” effect from images, several studies [13,14,15] have applied similar principles to UIE. Chiang et al. [14] achieved color enhancement of underwater images by compensating for color channels through a model that accounts for the attenuation of light energy with depth. Similarly, the Underwater Dark Channel Prior (UDCP) algorithm [15] enhances underwater images by ignoring the red channel and relying on information from the blue-green channels. Although physical model-based UIE methods have achieved some success in improving degraded image quality, they tend to exhibit weak generalization performance when dealing with complex and variable underwater environments.

### 2.2. Non-Physical Model-Based Methods

Unlike physical model-based methods, non-physical model-based methods typically enhance underwater images by directly adjusting pixel values. For example, Garg et al. [16] applied the Contrast Limited Adaptive Histogram Equalization (CLAHE) algorithm to enhance underwater images. Hu et al. [17] adopted an adaptive color correction method, the CLAHE algorithm, and multi-scale brightness fusion technology to enhance the details and contrast of images. Iqbal et al. introduced the UCM algorithm [18], which first enhances colors in the RGB color space and then adjusts contrast in the HSV color space to achieve image color correction and enhancement. However, methods relying on fixed parameters or heuristic rules often introduce issues such as overcompensation, color distortion, or insufficient detail processing in image reconstruction. Therefore, more adaptive and intelligent algorithms are needed to meet the challenges of UIE.

### 2.3. Deep Learning-Based Methods

With the rapid development of deep learning technologies, the field of image enhancement has seen new opportunities. Deep learning models, through large-scale training data, can learn deep features and structural information of images, making them more adaptive and robust in handling complex and dynamic environments. Among them, image enhancement techniques based on Convolutional Neural Networks (CNNs) and Generative Adversarial Networks (GANs) [19] have demonstrated outstanding performance in various image-processing tasks. Among CNN-based image enhancement applications, Li et al. [20] proposed a CNN-based image enhancement model, named UWCNN, which utilizes a synthetic database for training and improves the visibility of underwater images through an end-to-end data-driven mechanism. Saleh et al. [21] introduced an unsupervised UIE framework (UDnet), which employs a conditional variational autoencoder combined with probability-adaptive instance normalization and a statistically guided multi-color space stretching method to generate realistic underwater images. On the other hand, in the application of image enhancement using GAN as well as Cycle Consistent Generative Adversarial Networks [22] (CycleGAN). Li et al. [23] developed an enhancement model based on CycleGAN (SSIMCycleGAN). This model calculates the Structural Similarity Index (SSIM) between degraded and generated images and integrates it into the loss function, thereby improving the contrast of the generated images. Another model, SESSCycleGAN [24] employs the Nobuyuki Otsu method (OTSU) [25] to extract edge images from both the degraded image and generated high-quality underwater images and constrains the model by calculating the L1 distance between the two edge images. In addition, Bakht et al. [26] integrate multi-level attention mechanisms within the GAN architecture (MuLAGAN), enhancing the model’s ability to capture the details of underwater images. Cong et al. [27] proposed a physics model-guided GAN model (PUGAN) that adopts a dual-discriminator scheme to generate images that are both realistic and visually comfortable.

## 3. Materials and Methods

### 3.1. Architecture of LM-CycleGAN

**Figure 1:**
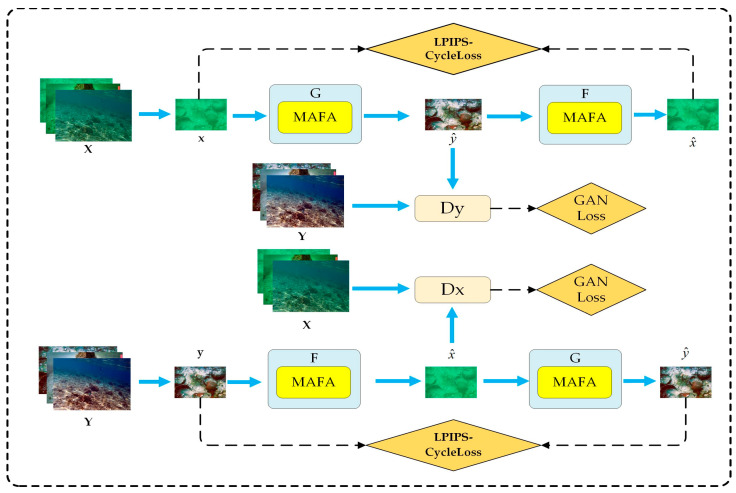
Network structure of LM-CycleGAN. X denotes the underwater degraded image domain and Y denotes the underwater high-quality image domain.

As illustrated in Figure 1, the LM-CycleGAN model consists of two sets of generators and discriminators. Generators G and F share the same network architecture as the discriminators $D_{X}$ and $D_{Y}$. Specifically, generator G transforms input images from the source domain X to the target domain Y, while generator F maps images from Y back to source domain X. In the discriminator module, $D_{X}$ and $D_{Y}$ are used to distinguish between real and generated images in domains X and Y, respectively. During the training process, a degraded image x is processed by generator G to produce a high-quality image $\hat{y}$. This image $\hat{y}$ is then combined with a randomly sampled real image y from the domain Y and fed into discriminator $D_{Y}$ for real-fake discrimination, generating the GAN Loss based on the discriminator’s output. The objective of the generator G is to maximize this loss, while the discriminator aims to minimize it. This adversarial training encourages the generator to produce images that closely resemble real images. Subsequently, $\hat{y}$ is passed through generator F to reconstruct the underwater degraded image $\hat{x}$. This reconstructed image is compared to the original x to compute the consistency loss. This process can be analogized to text translation tasks: Translate a sentence from Chinese to French, then back to Chinese. Compare the original sentence with the retranslated version to identify any differences. Enhance the performance of the translation system by minimizing these differences. Additionally, the literature [22] indicates that employing cycle consistency loss effectively mitigates the “mode collapse” problem, where the generator produces the same image no matter what the input image is.

To enhance the model’s ability to capture and restore image details, this study integrates the MAFA module into CycleGAN’s generator. Additionally, to ensure consistency between the reconstructed and original images, the LPIPS–Cycle Loss is employed. The LPIPS–Cycle Loss ensures that the reconstructed images $\hat{x}=F(G(x))$ and $\hat{y}=G(F(y))$ are as consistent as possible with the original input images x and y, respectively. By incorporating the LPIPS Loss, the model captures semantic similarities between images more effectively, compensating for the limitations of traditional L1 loss in capturing high-level visual information, thus significantly improving the perceptual quality of the generated images.

### 3.2. Generator Structure Based on MAFA

**Figure 2:**
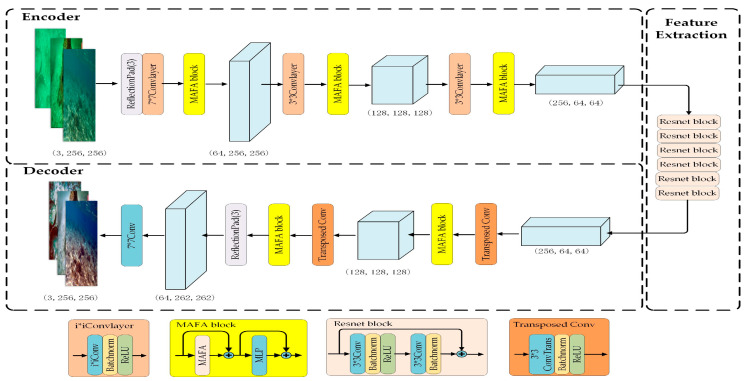
The network structure of the LM-CycleGAN generator, where the MLP is the Multi-Layer Perceptron, “n*nConv” denotes an operation that involves processing with a single convolutional kernel, and “⊕” denotes element-wise addition.

As illustrated in Figure 2, this study adopts the generator network architecture proposed by Johnson et al. [28], which consists of three convolutional layers, six residual blocks, three transposed convolutional layers, two ReflectionPad layers, and a convolutional layer that maps features to RGB. A key distinction of our approach is the incorporation of the efficient and robust feature extraction module MAFA into the backbone of the generator. The MAFA mechanism facilitates the adaptive fusion of multi-scale features, thereby effectively capturing details at various levels. This capability is particularly important for enhancing edge details in blurred underwater images.

**Figure 3:**
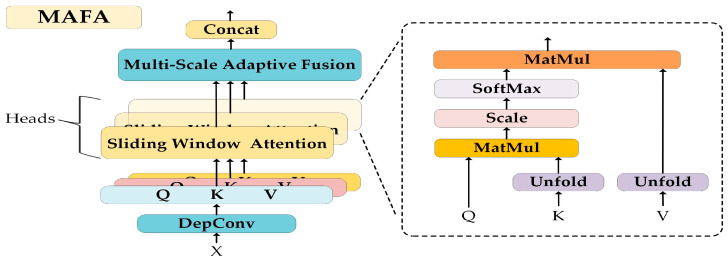
Network structure of Multi-scale Adaptive Fusion Attention (MAFA), where the dilation rates are set to [1,2,3,4], and the number of ‘heads’ is set to 8.

Building upon the Multi-Scale Dilated Attention mechanism (MSDA) [29], this paper introduces a novel Multi-scale Adaptive Fusion Attention mechanism (MAFA). Specifically, the mechanism first utilizes depthwise separable convolutions (DepConv) to transform the input feature maps, generating Query (Q), Key (K), and Value (V) feature maps. Thereafter, these feature maps are divided into different “heads” along the channel dimension. Each “head” employs the ‘nn.Unfold’ operation from PyTorch to perform sliding window attention at varying dilation rates, thus capturing local information at multiple scales. Additionally, each “head” is assigned a learnable weight vector. Following independent attention computations for each head, the output feature maps are weighted by their respective weight vectors. Finally, all weighted feature maps are aggregated along the channel dimension to form a comprehensive feature map. The design of the MAFA mechanism enables the model to dynamically adjust the importance of each head during training, effectively facilitating the adaptive fusion of features across different scales. Figure 3 illustrates the specific structure of the MAFA module.

### 3.3. Discriminator Network Structure

**Figure 4:**
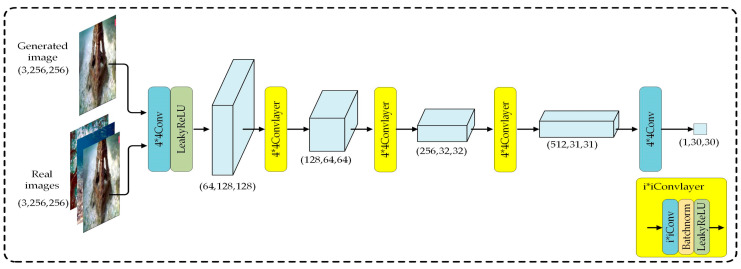
Network structure of LM-CycleGAN discriminator. Assuming the input is an RGB image with dimensions 3 × 256 × 256 pixels, the final output will be a tensor of size 1 × 30 × 30.

The network architecture of the LM-CycleGAN discriminator adopts the PatchGAN [30] structure, which is a fully convolutional network. Unlike traditional GAN discriminators that map the entire input image to a single probability value to determine whether it is real, PatchGAN employs a more localized and detailed evaluation strategy. It processes the input image through a fully convolutional network, ultimately producing an N × N feature map, where each element corresponds to a small patch of the input image. The value of each element reflects the likelihood that the corresponding patch belongs to a real image. This approach enables PatchGAN to track and quantify the realism of specific regions in the image, influencing the overall discrimination decision. The architecture of the LM-CycleGAN discriminator network, as shown in Figure 4, comprises five convolutional layers.

### 3.4. Loss Function

In LM-CycleGAN, the design of adversarial loss (GAN Loss) and cycle consistency loss (Cycle Loss) is consistent with the loss functions presented in CycleGAN [22]. To further enhance the model’s performance, structural consistency loss (LPIPS Loss) has also been introduced in this study to improve the perceptual quality and detail preservation of the generated images.

(1) GAN Loss

The GAN Loss consists of two parts: the forward process, which generates a high-quality image from a low-quality underwater image, and the reverse process, which generates a low-quality image from a real high-quality underwater image. The formula for the GAN Loss is given in Equation (1):

(1) $${GAN}_{Loss}=L_{GAN}(G,D_{Y},X,Y)+L_{GAN}(F,D_{X},Y,X)$$

For the forward process, the GAN Loss function is expressed as

(2) $$L_{GAN}(G,D_{Y},X,Y)==E_{y\sim P_{data(Y)}}[(D_{Y}(y)-1{)}^{2}]+E_{x\sim P_{data(X)}}[D_{Y}(G(x){)}^{2}]$$

For the inverse process, the adversarial loss function is expressed as

(3) $$\left(F,D_{X},Y,X)==E_{x\sim P_{data(X)}}[(D_{X}(x)-1{)}^{2}]+E_{y\sim P_{data(Y)}}[D_{X}(F(y){)}^{2}\right]$$

(2) Cycle Loss

The Cycle Loss is designed to ensure that the image is successfully returned to the original domain after being transformed through the two generators, thus ensuring the stability and accuracy of the generated image. The loss function for this part is denoted as:

(4) $$L_{cyc}\left(G,F\right)=E_{x\sim P_{data(x)}}[{\left\|F(G(x))-x\right\|}_{1}]+E_{y\sim P_{data(y)}}[{\left\|G(F(y))-y\right\|}_{1}]$$

where ${\left\|∙\right\|}_{1}$ denotes the L1 norm, which is used to calculate the pixel-by-pixel difference between two images.

(3) LPIPS Loss

**Figure 5:**
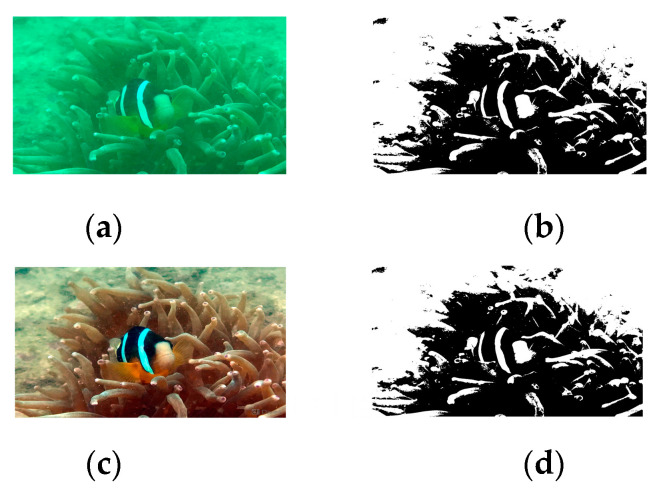
Original image and its edge image and generated image and its edge image: (**a**) Underwater degraded image; (**b**) Edge image corresponding to the underwater degraded image; (**c**) Generated underwater high-quality image; (**d**) Edge image corresponding to the underwater high-quality image.

In CycleGAN, the Cycle Loss calculates the similarity between two images using the L1 distance, which computes pixel-wise differences without accounting for the structural information between the images. To address this limitation, SESS-CycleGAN introduces edge constraints during the generation process from the source domain to the target domain. This approach utilizes the OTSU algorithm to obtain the edge images of both the original and generated images with the expectation of minimizing the L1 distance between them. The edge images corresponding to both the original and generated images are shown in Figure 5.

**Figure 6:**
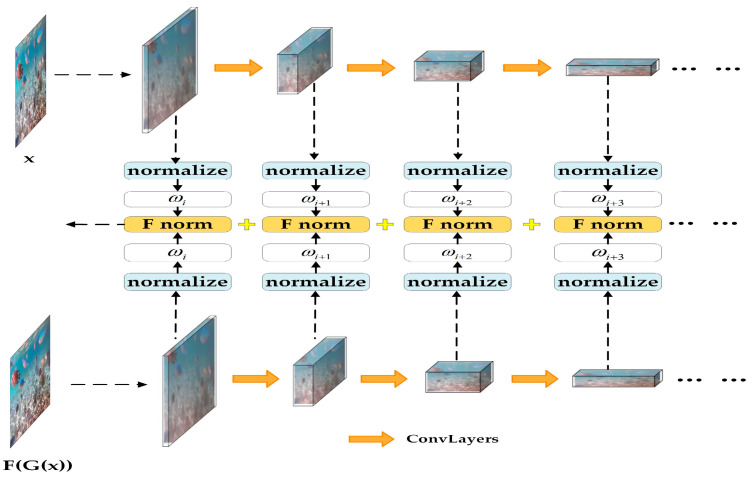
The network structure of Learned Perceptual Image Patch Similarity (LPIPS), $\omega_{i}$ denotes the specific weight layer corresponding to the ith output layer.

By adopting this strategy, structural information can be integrated into the CycleGAN loss function, ensuring it does not negatively impact color correction. However, the OTSU algorithm is sensitive to image noise, and its performance may be affected when there is a significant size disparity between the target and background or when dealing with multi-target underwater images. For instance, in Figure 5b,d, the edges of the fish blend with those of other elements, such as coral, leading to information loss. Since the presence of fish and vegetation is common in underwater tasks, the current method still faces limitations when handling complex scenes. To more effectively incorporate structural information into the training process of CycleGAN, this paper integrates LPIPS into the loss function. The architecture of LPIPS is shown in Figure 6.

The computation steps for the LPIPS [6] metric are as follows: First, the two images to be compared are passed through a pre-trained feature extraction network (such as AlexNet [31], SqueezeNet [32], or VGG [33]) to obtain feature representations. Then, the feature maps output by each layer are normalized along the channel dimension, with the normalized feature maps denoted as $x_{i}^{*}$ and ${F(G(x))}_{i}^{*}$, respectively. Next, the feature map is scaled using a specific weight layer. Following this, the Frobenius norm between the scaled feature maps is computed and averaged over the spatial dimensions. Finally, the Frobenius norms from all layers are summed to provide a similarity score, where smaller values indicate higher similarity. This process can be expressed by the following equation:

(5) $$LPIPS(x,\;F(G(x)))=\sum_{i}\frac{1}{H_{i}W_{i}}{\left\|\omega_{i}⊙(x_{i}^{*}-{F(G(x))}_{i}^{*})\right\|}_{F}$$

Here, $H_{i}$ and $W_{i}$ represent the corresponding width and height of the i-th layer feature map, $\omega_{i}$ represents the corresponding specific weight layer of the i-th layer, and ⊙ multiplies each feature map by the corresponding $\omega_{i}$. When denoting a matrix as A ϵ $R^{m\times n}$, the formula for calculating the Frobenius norm is as follows:

(6) $${\left\|A\right\|}_{F}=\sqrt{\sum_{i=1}^{m}\sum_{j=1}^{n}{\left|a_{ij}\right|}^{2}}$$

In this paper, AlexNet is selected as the feature extraction network. During the forward pass of CycleGAN, the original image x and the generated image F(G(x)) are input into the LPIPS network for similarity computation, with the generators G and F aiming to minimize Equation (5). This strategy introduces structural information during the model training process, further enhancing the performance of UIE. The loss function for this component is expressed as follows:

(7) $$L_{lpips}(G,F)=LPIPS(x,F(G(x)))+LPIPS(y,G(F(y)))$$

(4) Total loss function of LM-CycleGAN

In summary, the total loss function of LM-CycleGAN is expressed as

(8) $$L(G,F,D_{X},D_{Y})=L_{GAN}(G,D_{Y},X,Y)+L_{GAN}(F,D_{X},Y,X)+\alpha L_{cyc}(G,F)+\beta L_{lpips}(G,F)$$

where ‘α’ and ‘β’ represent the weight factors of Cycle Loss and LPIPS Loss, respectively. These factors are determined through hyperparameter tuning. In this paper, ‘α’ is set to 10, and ‘β’ is set to 5.

## 4. Experimental Results and Analysis

To comprehensively evaluate the proposed image enhancement model from multiple perspectives, Section 4.4 and Section 4.5 of this paper present a comparative analysis between LM-CycleGAN and other UIE algorithms, including traditional algorithms such as UDCP [15], CLAHE [16], and UCM [18], as well as deep learning-based methods such as UWCNN [20], UDnet [21], CycleGAN [22], SSIM-CycleGAN [23], SESS-CycleGAN [24], MuLA-GAN [26], and PUGAN [27]. In addition, the ablation study and real-time performance analysis are conducted in Section 4.6 and Section 4.7, respectively.

### 4.1. Dataset Introduction

**Figure 7:**
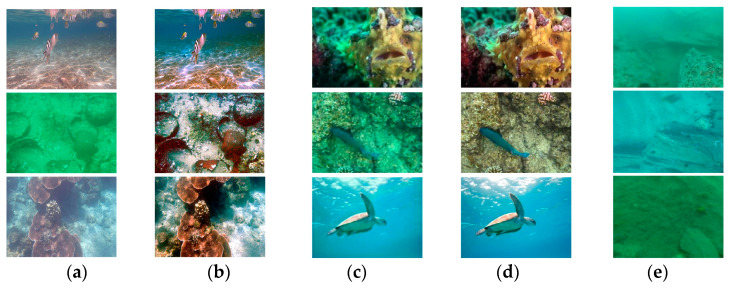
Sample images from the UIEB, EUVP, and RUIE datasets: (**a**,**b**) underwater degraded images and their corresponding reference images from the UIEB dataset; (**c**,**d**) underwater degraded images and their corresponding reference images from the EUVP dataset; (**e**) underwater degraded images from the RUIE dataset.

The UIEB dataset consists of 890 paired images divided into 800 training pairs and 90 test pairs. The EUVP test set consists of 100 paired images randomly selected from the EUVP-515 dataset. The RUIE test set consists of 90 challenging, no-reference, underwater degraded images characterized by images with blue, green, and blue-green casts. For a fair comparison, all models were trained on the UIEB dataset and tested on the UIEB, EUVP, and RUIE datasets (see Figure 7).

### 4.2. Experimental Settings

The experiments were conducted in a Windows 11 environment using a server manufactured by VirtAITech (Shanghai, China), which is equipped with an NVIDIA GeForce RTX 4090 GPU with 24 GB of VRAM. The deep learning framework employed was PyTorch 2.1.0, along with Python 3.9, PyCharm 2023, and CUDA version 11.8. The network was trained for 300 epochs on the UIEBD datasets, with a batch size of 1. The Adam optimizer was utilized, with an initial learning rate of 0.0002 and an exponential decay rate that reduced the learning rate to 0 after 150 epochs.

### 4.3. Evaluation Indicators

In the evaluation process, five commonly used metrics in UIE were employed to assess the quality of underwater reconstructed images: the Underwater Color Image Quality Evaluation (UCIQE) [34], the Underwater Image Quality Measurement (UIQM) [35], the Structural Similarity Index Measure (SSIM), Peak Signal-to-Noise Ratio (PSNR), and Average Gradient (AG).

UCIQE is a linear combination of chroma, contrast, and saturation values. UIQM is a linear combination of underwater image colorfulness measure, underwater image sharpness measure, and underwater image contrast measure. Higher scores for both UCIQE and UIQM indicate better performance in these aspects. SSIM quantifies the similarity between two images in terms of luminance, contrast, and structural information, with higher SSIM values indicating greater similarity. PSNR measures the ratio between the peak signal power and the noise power. Higher PSNR values indicate less image distortion and better quality. AG is calculated based on the mean gradient of an image. Higher AG values represent images with sharper edges and finer details.

Among these metrics, SSIM and PSNR are full-reference measures, requiring a comparison between the generated high-quality underwater image and the corresponding ground-truth image. In contrast, UCIQE, UIQM, and AG are no-reference metrics, which can be directly calculated from the images without the need for external reference images.

### 4.4. Visual Comparison with Other Methods

**Figure 8:**
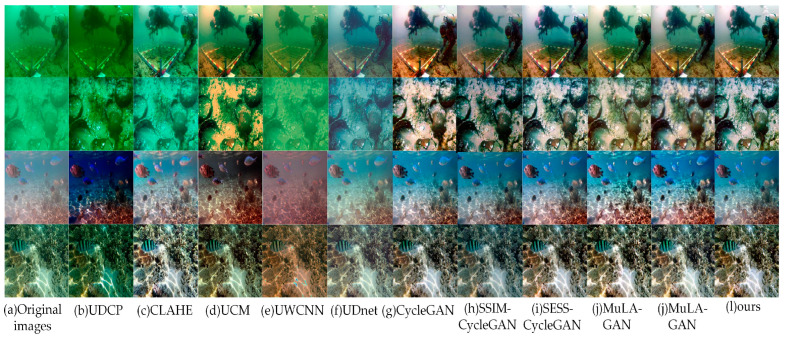
Visual comparison of image enhancement algorithms on the UIEB dataset.

**Figure 9:**
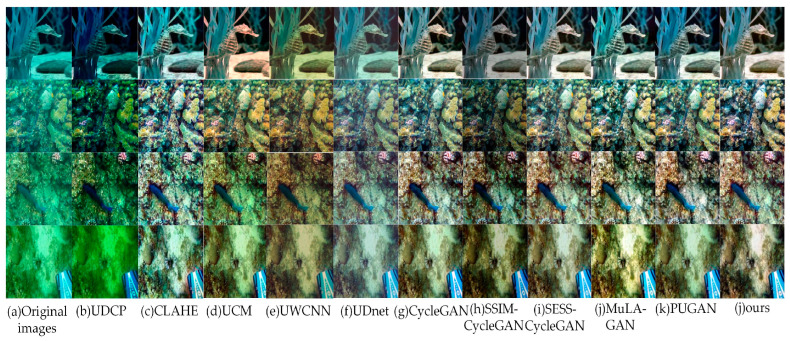
Visual comparison of image enhancement algorithms on the EUVP dataset.

**Figure 10:**
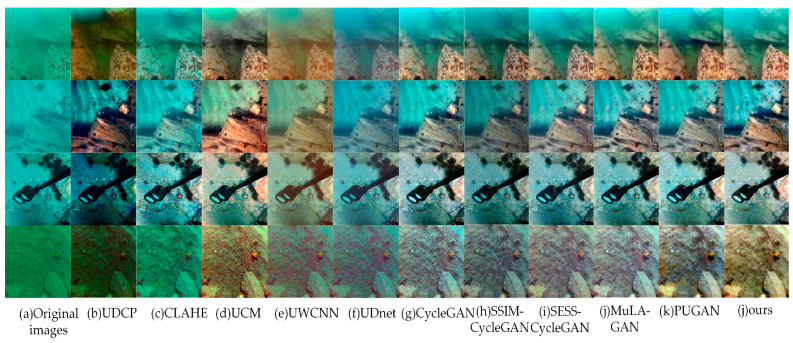
Visual comparison of image enhancement algorithms on the RUIE dataset.

Different UIE algorithms were evaluated on the UIEB, EUVP, and RUIE test sets, with the results presented in Figure 8, Figure 9 and Figure 10, respectively.

From Figure 8, Figure 9 and Figure 10, the UDCP, CLAHE, and UCM algorithms enhance image contrast to some extent. However, their performance in color correction is not particularly impressive. The processed images still display significant color distortion and fail to effectively eliminate the “haze”. In contrast, GAN-based algorithms excel in color correction, accurately matching the color distributions of the target images. Nevertheless, images enhanced by CycleGAN exhibit localized red checkerboard artifacts and blurred edge details, which may be attributed to the limited ability of the CycleGAN generator to extract detailed features from images. Images processed by SSIM-CycleGAN tend to be darker, likely due to its requirement for consistency in SSIM metrics (brightness, contrast, and structural information) between the original and generated images while overlooking the inherent brightness differences that exist between them, resulting in unintended negative effects. The SESS-CycleGAN, MuLA-GAN, PUGAN, and the method proposed in this paper all achieve satisfactory results on the UIEB and EUVP datasets, with only slight differences in color distribution at the subjective visual level. Notably, on the challenging no-reference RUIE dataset, the images generated by our proposed method exhibit higher contrast and improved clarity. This indicates that our model demonstrates exceptional capability in capturing image details and possesses stronger generalization performance.

### 4.5. Objective Comparison with Other Methods

Table 1, Table 2 and Table 3 present the performance metrics of various UIE methods on the UIEB, EUVP, and RUIE datasets, respectively. Values in bold indicate the best performance, and values underlined indicate the second-best performance.

By analyzing the experimental data shown in Table 1, Table 2 and Table 3, the proposed LM-CycleGAN algorithm achieved the best scores in SSIM, PSNR, and AG metrics. This suggests that the algorithm can not only accurately restore the details of target images, and reduce image distortion and noise, but also enhance visual quality, improving clarity and contrast. In the UCIQE and UIQM metrics, the UDCP algorithm achieved the optimal value, while the CLAHE algorithm and the UCM algorithm achieved the sub-optimal value. When considering the subjective visual representations provided in Section 4.4, conclusions similar to those found in the literature [9], ref. [36] can be drawn. Given that the UCIQE and UIQM metrics primarily focus on linear combinations of low-level features such as contrast and saturation, they neglect higher-level semantic information or prior knowledge related to human visual perception. Additionally, these measures do not evaluate whether the intensity values of the entire image fall within a reasonable range. Consequently, although the UDCP, CLAHE, and UCM algorithms score higher in UCIQE and UIQM values, there is still a significant gap between the image quality produced by these algorithms and human visual perception. Notably, compared to deep learning-based methods, the proposed LM-CycleGAN algorithm not only achieved optimal values in these two metrics but also generated images that better align with human visual perception.

### 4.6. Ablation Experiment

A series of ablation experiments were conducted to evaluate the effectiveness of the components in the proposed LM-CycleGAN model. Table 4 presents the performance comparison between the traditional CycleGAN model and the models augmented with different improvement modules. These improvement models include the CycleGAN model integrating the MSDA mechanism into the generator structure (hereafter referred to as MSDA), the CycleGAN model embedding the MAFA mechanism into the generator structure (hereafter referred to as MAFA), the CycleGAN model incorporating LPIPS Loss (hereafter referred to as LPIPS), the CycleGAN model with the joint MSDA mechanism and LPIPS Loss (hereafter referred to as MSDA + LPIPS), and the CycleGAN model with the joint MAFA mechanism and LPIPS Loss (the proposed methods).

Table 4 shows that compared to the control group (T1), groups T2, T3, and T4 exhibit significant improvements across all five key performance metrics. This indicates that introducing the MSDA mechanism, MAFA mechanism, and LPIPS Loss effectively suppresses noise while revealing richer and more intricate texture details. Furthermore, the comparison between groups T2 and T3 demonstrates the effectiveness of the multi-scale feature adaptive fusion strategy. The results for groups T5 and T6 indicate that the combined model of MAFA and LPIPS outperforms the combined model of MSDA and LPIPS, further underscoring the superiority of the MAFA mechanism. Overall, the LM-CycleGAN model that integrates MAFA and LPIPS strategies (group T6) consistently outperforms the other models across all five evaluation metrics.

**Figure 11:**
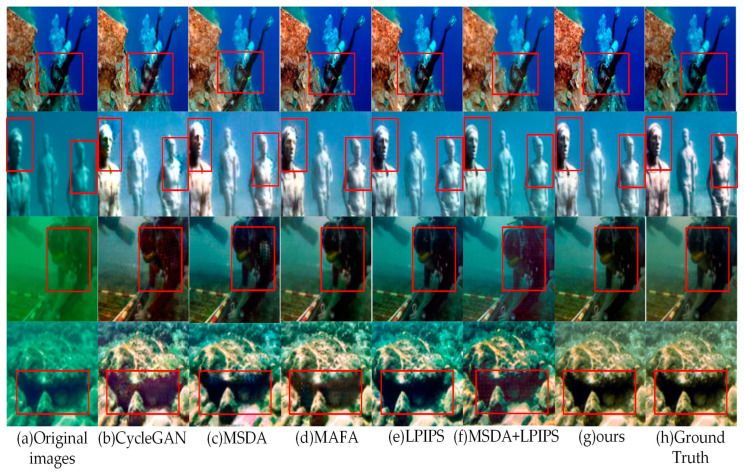
Comparison of enhancement effects from different strategies on the UIEB dataset, where the red box highlights local enhancement areas.

Figure 11 demonstrates the image enhancement effects of various improvement models on the UIEB dataset. Traditional CycleGAN-reconstructed images exhibit significant red checkerboard artifacts and blurred edge details. Images processed with MSDA have partially eliminated the red artifact but still exhibit color bias, localized detail blur, and slight grid effects. In contrast, images processed with MAFA have more realistic colors but still exhibit issues with localized detail blur. Images processed with LPIPS demonstrate clearer edge details, although they still present some color bias. The model that unites MAFA and LPIPS has made improvements in addressing localized detail blur but still has room for enhancement in color correction. Notably, the LM-CycleGAN model achieved the desired effects: images processed by it not only eliminated the red checkerboard artifacts and enhanced the clarity of texture details in the reconstructed underwater images but also maintained color accuracy.

### 4.7. Real-Time Analysis and Discussion

Given that UIE techniques are typically deployed on resource-constrained devices, this section will examine and analyze the real-time performance of 11 UIE methods. To better simulate the real-world effects of deploying UIE technologies on such devices, we chose the relatively low-performance NVIDIA GeForce MX130 GPU for this experiment. In this analysis, three key performance indicators are considered: parameters, FLOPs, and FPS. Parameters refer to the number of trainable variables in a model, FLOPs (Floating Point Operations Per Second) indicate the computational complexity of a model, and FPS (Frames Per Second) measures the number of frames processed in one second, reflecting the real-time performance of an image processing method.

Through the analysis of Table 5, traditional UIE algorithms exhibit fast processing speeds (FPSs) due to their direct application of fixed rules or mathematical formulas for image processing. Among deep learning methods, although UWCNN has the smallest network size, its real-time performance is compromised as it requires physical models to generate additional images. MuLA-GAN achieves the highest FPS with fewer parameters and lower computational complexity. In contrast, the method proposed in this paper achieves an FPS of 94.3. Although this is not the optimal performance, it is sufficient to meet the real-time requirements for underwater operations.

## 5. Conclusions

This paper proposes an advanced UIE method, LM-CycleGAN, which has achieved excellent experimental results on publicly available UIEB, EUVP, and RUIE datasets. By applying the multi-scale feature adaptive fusion strategy, LM-CycleGAN can effectively capture important detail features in underwater images, thereby enhancing the model’s adaptability in diverse environments. Additionally, by introducing LPIPS Loss, the model pays more attention to structural consistency during the reconstruction process, which further enhances the ability to recover details in complex underwater images. However, the model performs poorly when handling high-saturation blue and green images, which are very common in underwater environments. A possible reason for this limitation is the insufficient number of such samples in the training dataset, which restricts the model’s generalization ability in these scenarios, leading to issues such as color bias and detail loss. Future research will further explore directions such as dataset augmentation and model lightweight to provide more robust and efficient solutions for underwater image enhancement.

## Figures and Tables

**Table 1:** Performance comparison of various algorithms on the UIEB dataset (**↑** indicates that higher values are more desirable).

Model/Method	UCIQE ↑	UIQM ↑	SSIM ↑	PSNR ↑	AG ↑
UDCP	**0.5230**	**1.3371**	0.6439	28.2402	9.8035
CLAHE	0.4429	1.029 3	0.7190	28.2846	10.5692
UCM	0.4927	1.0138	0.7177	28.6008	10.9695
UWCNN	0.3707	0.5164	0.7066	28.0678	6.7591
UDnet	0.3554	0.4492	0.7665	28.1062	7.5259
CycleGAN	0.4504	0.7244	0.7554	28.2691	11.1365
SSIM-CycleGAN	0.4618	0.8319	0.7717	28.9576	11.3564
SESS-CycleGAN	0.4608	0.8330	0.7593	28.8892	11.3594
MuLA-GAN	0.4542	0.8325	0.7759	28.8459	10.0260
PUGAN	0.4628	0.7921	0.7714	28.9979	9.2452
ours	0.4842	0.8936	**0.7933**	**29.2120**	**11.6876**

**Table 2:** Performance comparison of various algorithms on the EUVP dataset (**↑** indicates that higher values are more desirable).

Model/Method	UCIQE ↑	UIQM ↑	SSIM ↑	PSNR ↑	AG ↑
UDCP	**0.5204**	**1.1924**	0.5799	28.0624	7.3796
CLAHE	0.4597	1.0586	0.7295	28.3214	8.3591
UCM	0.4745	0.9554	0.7573	28.6456	8.6528
UWCNN	0.3958	0.5329	0.7437	28.0347	6.0778
UDnet	0.3649	0.4618	0.7535	28.1286	6.0875
CycleGAN	0.4457	0.7643	0.7635	28.2800	9.0708
SSIM-CycleGAN	0.4482	0.7989	0.7771	28.4973	9.5739
SESS-CycleGAN	0.4454	0.7775	0.7747	28.6656	9.4611
MuLA-GAN	0.4571	0.7819	0.7730	28.4388	9.9494
PUGAN	0.4495	0.8178	0.7647	28.8436	9.0193
ours	0.4669	0.8358	**0.7883**	**29.1524**	**10.7343**

**Table 3:** Performance comparison of various algorithms on the RUIE dataset (**↑** indicates that higher values are more desirable).

Model/Method	UCIQE ↑	UIQM ↑	AG ↑
UDCP	**0.4227**	**0.9736**	6.1208
CLAHE	0.3409	0.4619	7.8120
UCM	0.3932	0.7004	8.6002
UWCNN	0.3707	0.5164	6.7591
UDnet	0.3275	0.3614	4.5951
CycleGAN	0.3618	0.5765	7.8345
SSIM-CycleGAN	0.3638	0.6150	8.2624
SESS-CycleGAN	0.3627	0.6209	8.4896
MuLA-GAN	0.3688	0.6394	8.3231
PUGAN	0.3715	0.6288	8.7618
ours	0.3890	0.6569	**8.9634**

**Table 4:** Performance evaluation of CycleGAN models with various improvements on UIEB (**↑** indicates that higher values are more desirable and “√” indicates that the corresponding module has been added to CycleGAN).

Experiments	MSDA	MAFA	LPIPS	UCIQE ↑	UIQM ↑	SSIM ↑	PSNR ↑	AG ↑
T1	—	—	—	0.4504	0.7244	0.7554	28.2691	11.1365
T2	√	—	—	0.4534	0.7708	0.7661	28.6008	11.3141
T3	—	√	—	0.4587	0.8491	0.7765	29.0604	11.4885
T4	—	—	√	0.4629	0.8501	0.7839	28.9574	11.4448
T5	√	—	√	0.4784	0.8763	0.7896	29.9013	11.5635
T6	—	√	√	**0.4842**	**0.8936**	**0.7933**	**29.2120**	**11.6876**

**Table 5:** Real-time performance analysis of 11 underwater image enhancement methods (↑ indicates that higher values are more desirable, while ↓ is the opposite).

Model/Method	Params (M) ↓	FLOPs (G) ↓	FPS (Hz) ↑
UDCP	—	—	142.3
CLAHE	—	—	154.2
UCM	—	—	128.4
UWCNN	1.1	3.08	42.3
UDnet	16.1	96.3	54.3
CycleGAN	11.4	58.2	99.2
SSIM-CycleGAN	13.6	63.3	89.4
SESS-CycleGAN	13.1	62.7	90.9
MuLA-GAN	17.3	30.2	117.8
PUGAN	95.7	72.5	62.5
ours	12.6	60.1	94.3

## Data Availability

The data presented in this study are available on request from the corresponding author.
